# Minoxidil delivered via a stem cell membrane delivery controlled release system promotes hair growth in C57BL/6J mice

**DOI:** 10.3389/fbioe.2023.1331754

**Published:** 2024-01-08

**Authors:** Dandan Song, Shouxi Pan, Wenxia Jin, Ronghui Wu, Tianqi Zhao, Jinlan Jiang, Mingji Zhu

**Affiliations:** ^1^ Department of Dermatology, China-Japan Union Hospital of Jilin University, Changchun, China; ^2^ Norman Bethune College of Medicine, Jilin University, Changchun, China; ^3^ Lanzhou Institute of Biological Products Co., Ltd., Lanzhou, China; ^4^ Scientific Research Center, China-Japan Union Hospital of Jilin University, Changchun, China

**Keywords:** minoxidil, stem cell membrane, nanoparticle, hair follicle delivery, hair growth

## Abstract

**Objective:** Umbilical cord-derived mesenchymal stem cell membrane-loaded minoxidil (MXD) nanoparticles (STCM-MXD-NPs) were prepared to investigate their effects on hair growth in C57BL/6J mice.

**Methods:** STCM-MXD-NPs were obtained by freeze-thawing and differential centrifugation, and their effects on hair growth were evaluated using C57BL/6J mice. The mRNA and protein expression levels of vascular endothelial growth factor (VEGF) and insulin-like growth factor-1 (IGF-1) were detected by real-time polymerase chain reaction and enzyme-linked immunosorbent assays, respectively. Protein expression levels of marker of proliferation Ki-67 (MKI67) and β-catenin (CTNNB) in skin tissue were detected by immunohistochemistry.

**Results:** STCM-MXD-NPs improved MXD solubility. They released the drug slowly, increasing its transdermal properties, accumulation in the skin, and content in the hair bulb tissues with a better efficacy than that of ordinary MXD. Moreover, STCM-MXD-NPs significantly upregulated the mRNA and protein levels of VEGF and IGF-1 and promoted the protein expression of MKI67 and CTNNB in mouse skin tissues, promoting mouse hair growth.

**Conclusion:** Stem cell membrane-loaded MXD nanoparticles with slow-release properties increased MXD accumulation in the skin by improving its transdermal properties, increasing VEGF, IGF-1, MKI67, and CTNNB expression levels and promoting hair growth in C57BL/6J mice.

## Introduction

Alopecia is a common skin disease in which normally growing hairs are in the anagen phase. When the dynamic balance between the anagen and telogen phases is disrupted, the hairs enter the telogen phase prematurely, or the telogen phase is prolonged, resulting in alopecia. While alopecia does not cause direct physical harm, it seriously affects the patient’s psychology and quality of life and has become a chronic disease that urgently needs to be addressed (Arca et al., 2004; Varothai and Bergfeld, 2014; Juárez-Rendón et al., 2017; York et al., 2020; Jankowski and Frith, 2022).

With the incidence of alopecia increasing annually, the slow growth rate of hair, and the multiple side effects caused by therapeutic drugs leading to a significant psychological burden for patients with alopecia, its treatment has now become an important part of clinical dermatology (Vañó-Galván and Camacho, 2017; Ocampo-Garza et al., 2019). Among the drugs used to treat hair loss, minoxidil (MXD) is the most commonly used US Food and Drug Administration-approved topical drug for treating alopecia. It acts as a vasodilator of small arteries. It is metabolized to MXD sulfate by sulphotransferase in skin tissues, opening up the potassium ion channels and promoting the premature entry of resting hair follicles into the anagen phase by shortening the telogen phase and lengthening the anagen phase by increasing follicle size, promoting hair growth (Kiesewetter et al., 1991; Messenger and Rundegren, 2004; Goren et al., 2015; Barbareschi, 2018; Suchonwanit et al., 2019). However, due to its shortcomings of low absorption rate and long treatment period, only a few patients receive a good therapeutic effect. The MXD tincture irritates the skin and can cause some adverse reactions, making it difficult for patients to adhere to the treatment (Rossi et al., 2012; Randolph and Tosti, 2021; Kim et al., 2022).

Nano-drug delivery systems have remarkable controllability for precise and continuous drug release at the target site. Small particle size facilitates dermal drug delivery, penetrates deeper into the skin, and improves transdermal transport into and across the skin barrier for enhanced transdermal transport (Alvarez-Román et al., 2004; Ji et al., 2021). Our preliminary research found that cell membrane drug-carrying technology could be used to modify nanoparticles (NPs) so that they gain the functions and properties unique to cell membranes, improving their biocompatibility and targeting, with good transdermal, low irritation, and slow release properties (Wang et al., 2020).

**FIGURE 1:**
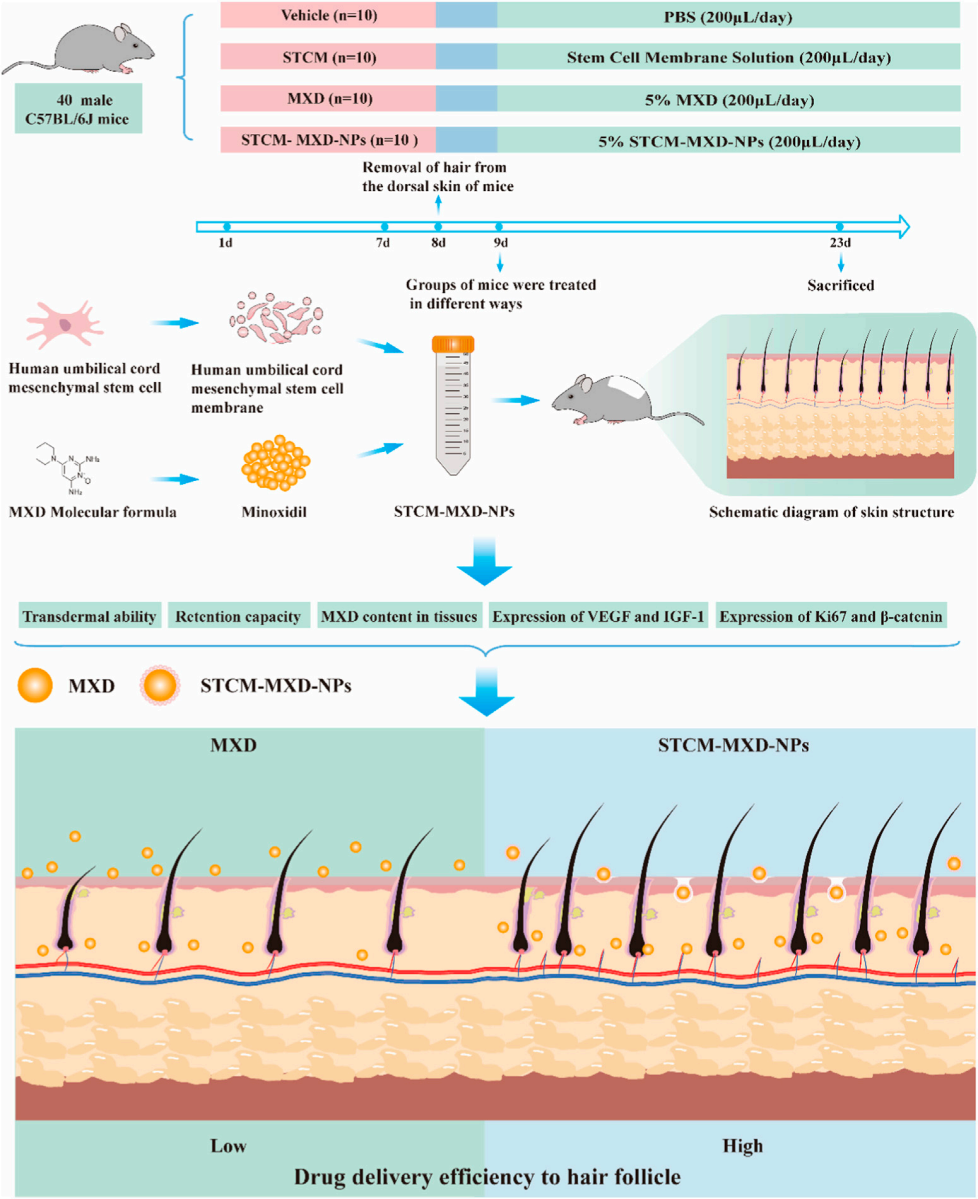
Animal experiment protocol and design.

In this study, we designed human umbilical cord-derived mesenchymal stem cell (hMSC) membrane (STCM)-loaded MXD NPs (STCM-MXD-NPs). We then used C57BL/6J mice as a hair growth model to assess the role of the stem cell membrane nano-loaded controlled-release system delivering MXD to promote hair growth (Figure 1).

## Materials and methods

### Cell lines and antibodies

The hMSCs were obtained from the Scientific Research Centre of China-Japan Union Hospital, Jilin University (Changchun, China). A vascular endothelial growth factor (VEGF) enzyme-linked immunosorbent assay (ELISA) kit was obtained from Cosmo Bio Co., Ltd. (Tokyo, Japan). A mouse/rat insulin-like growth factor-1 (IGF-1) ELISA kit was obtained from R&D Systems (Minneapolis, MN, United States). Immunohistochemical antibodies against marker of proliferation Ki-67 (MKI67) and β-catenin (CTNNB) were obtained from Abcam Tec (Shanghai, China). Minoxidil was obtained from Shanghai yuanye Bio-Technology Co., Ltd., (Shanghai, China).

### STCM-MXD-NP preparation

The hMSC membrane was prepared according to published protocols (Gao et al., 2016; Yao et al., 2023). Briefly, 4 × 10^6^ hMSCs were lysed with hypotonic solution, and purified stem cell membranes were obtained by freeze-thawing and differential centrifugation. Next, MXD was mixed with the hMSC membrane solution, placed in an ultrasonic cleaner for 10 min, and centrifuged at 10,000 g for 30 min. Then, the pellet was resuspended in phosphate-buffered saline (PBS), and the precipitate was the STCM-MXD-NPs.

The morphology of the nanoparticles was observed by transmission electron microscope (TEM; JEM-2100F, JEOL, Tokyo, Japan). The samples were dispersed directly into PBS. A drop of the STCM-MXD-NPs suspension was transferred to copper grid. After staining with 3% (w/v) phosphotungstic acid solution and drying at room temperature, the sample was observed by TEM.

### Ultraviolet spectrophotometry of STCM-MXD-NPs

The absorption spectra of hMSC membranes, MXD, and STCM-MXD-NPs were measured at the same concentration using a UV spectrophotometer at a 231 nm wavelength to verify whether the hMSC membranes were successfully encapsulated with MXD.

### High-performance liquid chromatography analysis of STCM-MXD-NPs

This analysis used an Agilent ZORBAX SB-C18 (4.6 × 250 mm; 5 μm) column, methanol-water (70:30) mobile phase, 1.0 mL/min flow rate, 285 nm detection wavelength, and 30°C column temperature. A 1 mL sample of MXD solution or stem cell membrane-loaded MXD nanoparticle solution was filtered through a 0.22 μm microporous filter membrane. Then, 20 μL of this renewed filtrate was injected into the high-performance liquid chromatography (HPLC) instrument. The samples’ peak times and shapes were compared to verify whether the hMSC membrane was successfully loaded with MXD (Wang et al., 2017).

### Particle size and zeta potential of STCM-MXD-NPs

The STCM-MXD-NPs were diluted with double-distilled water and then purified with a 220 nm filter membrane. Their particle distribution and zeta potential were measured with a laser diffraction size distribution analyzer and zeta potential meter.

### Determination of the encapsulation rate of STCM-MXD-NPs

The amount of unencapsulated MXD was determined at a 231 nm wavelength using a UV spectrophotometer. The encapsulation rate was calculated using the formula: encapsulation rate = (total drug amount − unencapsulated drug amount)/total drug amount.

### STCM-MXD-NP drug release assay

Briefly, 1 mL of STCM-MXD-NPs at 1 mg/mL was placed in a dialysis bag in the *in vitro* release medium and stirred uniformly at 200 rpm and a constant temperature of 37°C ± 0.5°C. Next, 1 mL of the release solution was removed at different time points, and the original solution was supplemented with an equal amount of media buffer. Then, the MXD concentration in the medium was determined by UV spectrophotometry, the cumulative percentage of drug release was calculated, and the release curve was plotted.

### STCM-MXD-NP transdermal assay

The treated experimental pig skin was placed between the two-halves of the Franz diffusion cell and secured with clamps in a circulating water bath with a rotational speed of 200 rpm and 32°C ± 0.5°C. Next, 1 mL each of pig skin grinding solution, stem cell membrane solution, 1 mg/mL of MXD, and STCM-MXD-NPs containing 1 mg/mL of MXD were added to the supply chamber, which was then sealed with cling film to prevent water evaporation. Then, 1 mL samples were taken from the receiving cell at 2, 4, 6, 8, 10, 12, and 14 h and replaced with an equal volume of fresh receiving solution at the appropriate temperature. After filtration through a 0.22 μm filter membrane, the sample’s drug content was determined by HPLC.

### STCM-MXD-NPs skin retention assay

At the end of the transdermal experiment, the pig skin was removed, rinsed 3–4 times with PBS buffer, and then cut into pieces. Next, 1 mL of saline was added, and the sample was homogenized. Then, MXD was extracted from the pig skin with anhydrous ethanol in three batches (1 mL was used each time). Next, the supernatant was centrifuged at 12,000 rpm for 30 min at a low temperature. Then, the drug concentration was determined using HPLC, and the amount of drug retention was calculated.

### Experimental animals

Forty specific-pathogen-free-grade C57BL/6J mice aged 7 weeks and weighing 18 ± 2 g were obtained from the Experimental Animal Centre of Jilin University. All mice were housed under standard conditions (room temperature: 25°C; light/dark time = 12/12 h). The experiments were conducted after 1 week of acclimatization, during which time the mice were supplied with adequate food and water, and the cages and bedding were cleaned every 2 days. The experimental protocol was approved by the Ethics Committee of Jilin University.

The mice were randomly divided into four groups of 10: vehicle (PBS), STCM, 5% MXD, and 5% STCM-MXD-NPs. The hair on the back of the mice was removed before the experiment. During the experiment, 200 μL of PBS, STCM, 5% MXD, or 5% STCM-MXD-NPs was evenly applied to the skin of the depilated area on the back of the mice over an area of about 2 cm × 2 cm. The treatment was administered once daily for 15 consecutive days.

### Observation of hair growth on the back of C57BL/6J mice

Hair growth was monitored daily at 9:00 a.m. using a digital camera under the same light source. Changes in hair area were assessed using the ImageJ image analysis software. The area under the hair area-time curve (AUC) was calculated.

### Histopathological observation of hair growth

After 15 days, the mice were euthanized by injection of a lethal pentobarbital dose. The skin was taken from the hair removal site on their back, fixed with 4% paraformaldehyde, dehydrated, embedded in paraffin wax, and stained with hematoxylin for 3–5 min. Next, the sections were dehydrated with various ethanol concentrations for 5 min each and stained with eosin. Then, the sections were dehydrated in rows, made transparent, and sealed with neutral gum. The hematoxylin-eosin (HE)-stained sections were observed under an ordinary microscope. The morphology of hair follicles was observed, the total number of hair follicles in each group was counted, and the ratio of anagen/telogen (A/T) follicles was determined.

### Measurement of MXD levels in skin tissue and hair bulbs

The mice were euthanized by injection of a lethal pentobarbital dose. Their hair was collected with forceps, and the hair bulb region was separated. Next, the hair bulb and the rest of the skin tissue were homogenized separately in ethanol, and the homogenate was centrifuged at 20,400 g for 20 min at 4°C. Then, the MXD content in the supernatant was measured by HPLC as previously described (Wang et al., 2017). In this study, the MXD contents in the skin tissue and hair bulb are expressed as per mg protein, with total protein measured using a Bio-Rad protein assay kit.

### Quantitative real-time polymerase chain reactions (qPCR)

The mice were euthanized by injection of a lethal pentobarbital dose. The total RNA in their hairballs was extracted using the Trizol reagent. The reverse transcription and qPCR reactions were performed using an RNA PCR Kit and LightCycler FastStart DNA Master SYBR Green I Kit according to the manufacturer’s instructions. RNA concentration and purity were determined using a Nanodrop 2000. A 20 μL reverse transcription reaction system was prepared to synthesize the complementary DNA (cDNA). Then, the cDNA was subjected to qPCR, with three replicate wells per sample. The qPCR procedure comprised pre-denaturation for 30 s, followed by 40 cycles of denaturation at 95°C for 15 s and annealing/extension at 60°C for 30 s. The mRNA expression levels of *Vegf* and *Igf-1* were calculated.

### Measurement of VEGF and IGF-1 proteins in hairballs

Mice were euthanized by injection of a lethal pentobarbital dose. Hairball samples were homogenized in purified water according to the manufacturer’s instructions and centrifuged at 20,400 g for 20 min at 4°C. Next, the homogenates were incubated in microtiter plate wells pre-packed with mouse monoclonal antibodies against IGF-1 and VEGF. Then, detection reagents were added, and the absorbance was measured at 450 nm using a microplate reader.

### Immunohistochemical staining

After paraffin sections were deparaffinized and washed with water, they were placed in a microwave oven for antigen repair before being left to cool naturally. Next, the sections were washed, incubated, and washed again for serum closure. Then, primary antibodies against MKI67 and CTNNB were added separately and incubated overnight at 4°C. Next, a secondary antibody was added. Then, the section was washed and developed using 3,3′-diaminobenzidine, with color development terminated by rinsing the sections with running water; positive cells stained brownish yellow. The sections were counterstained with hematoxylin and washed with distilled water. Finally, the sections were dehydrated to make them transparent, sealed, and observed and imaged under a light microscope.

### Statistical analysis

Data were statistically analyzed using SPSS software (version 19.0). Data are expressed as mean ± standard deviation (SD) and compared between groups using one-way analysis of variance (ANOVA). A *p* < 0.05 was considered statistically significant. The Origin software was used to create the graphs. Key: *, **, and *** represent *p* < 0.05, *p* < 0.01, and *p* < 0.001, respectively.

## Results

### STCM-MXD-NP synthesis and characterization

**FIGURE 2:**
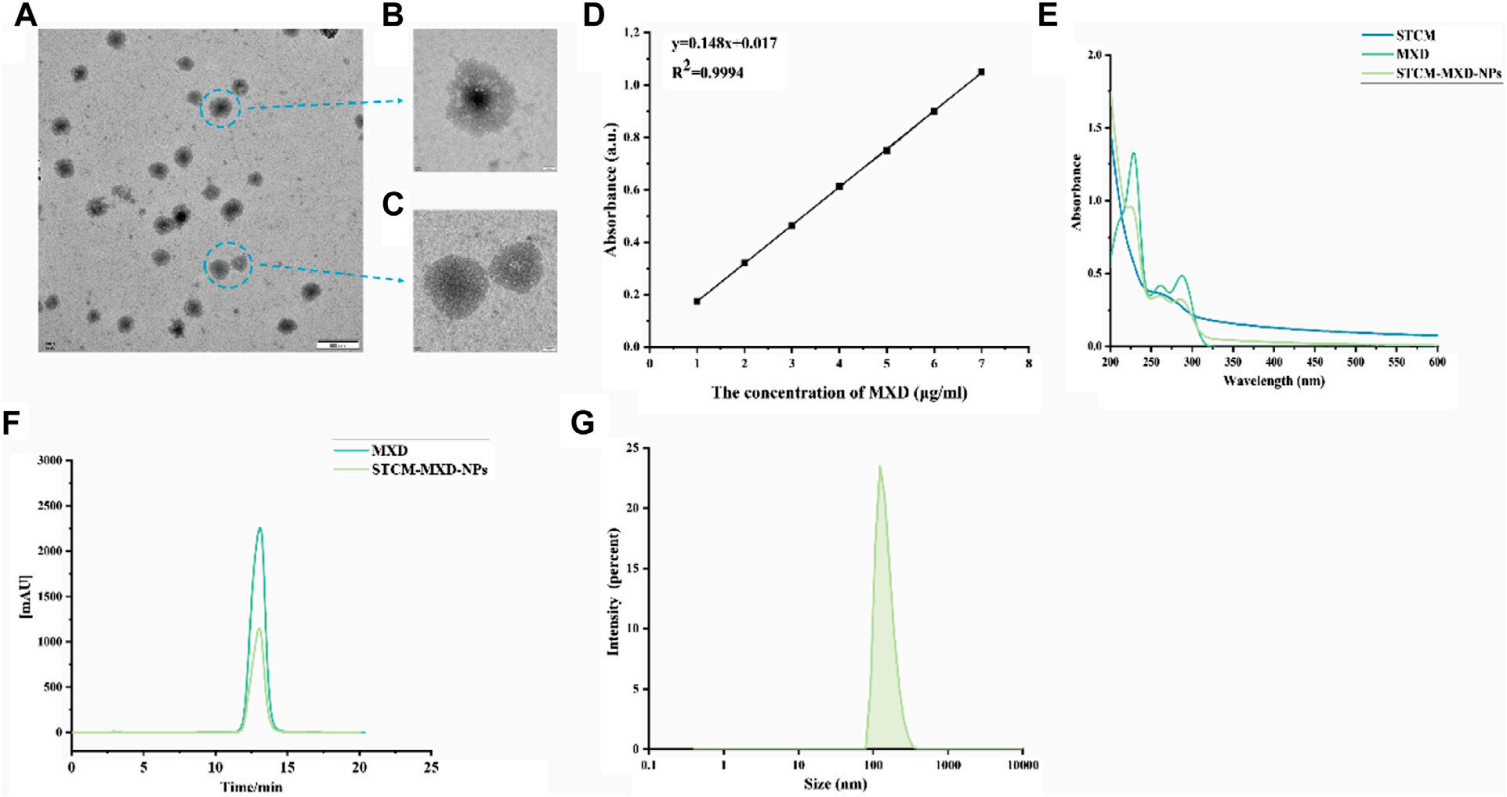
STCM-MXD-NP synthesis and characterization. **(A)** Morphology of STCM-MXD-NPs sample preparation by TEM (500 nm). **(B)** Morphology of STCM-MXD-NPs by TEM (50 nm). **(C)** Morphology of stem cell membrane by TEM(50 nm). **(D)** MXD standard curve. **(E)** The absorbance spectrum confirms STCM-MXD-NP synthesis. **(F)** HPLC confirmed the MXD peak-out time and STCM-MXD-NP synthesis. **(G)** Particle size distribution of the STCM-MXD-NPs.

The morphology of STCM-MXD-NPs captured under transmission electron microscope was shown in Figures 2A–C. The MXD standard had a characteristic absorption peak at 231 nm. Its standard curve of absorbance at 231 nm as a function of concentration is shown in Figure 2D (*y* = 0.1479x + 0.0166; *R* ^2^ = 0.9994), which shows a good linear relationship. The STCM-MXD-NP solution showed an absorption peak near 231 nm. The peak times of the STCM-MXD-NP and MXD solutions were the same, both at about 13 min, and the peaks had the same shape (Figures 2E, F), showing that the hMSC membranes were successfully encapsulated with MXD. In addition, we examined the zeta potential of the STCM-MXD-NPs. The newly prepared STCM-MXD-NPs were diluted and placed in a nanoparticle-size zeta potential meter. Their particle size was detected to be 181.4 ± 5.98 nm (Figure 2G), and their zeta potential was −31.6 mV.

The STCM-MXD-NPs prepared with different concentrations of MXD and hMSC membrane solutions showed different encapsulation efficiencies. The encapsulation rate first increased and then decreased with increasing MXD concentration, and the encapsulation rate reached about 62.56% at 1–9 mg/mL (Supplementary Data S1).

Then, we assessed MXD solubility by dissolving MXD in ethanol solution or PBS. MXD seemed insoluble in PBS, with white crystals gradually deposited at the bottom of the tube. However, it was soluble in ethanol. The hMSC membrane dissolved in PBS was a colorless transparent liquid. The STCM-MXD-NPs prepared by ultrasonication and low-temperature differential centrifugation formed a white precipitate. STCM-MXD-NPs dissolved in PBS formed a white suspension, indicating that hMSC membranes loaded with MXD NPs improved MXD solubility (Supplementary Data S2, S3).

### Sustained release of STCM-MXD-NPs *in vitro*

**FIGURE 3:**
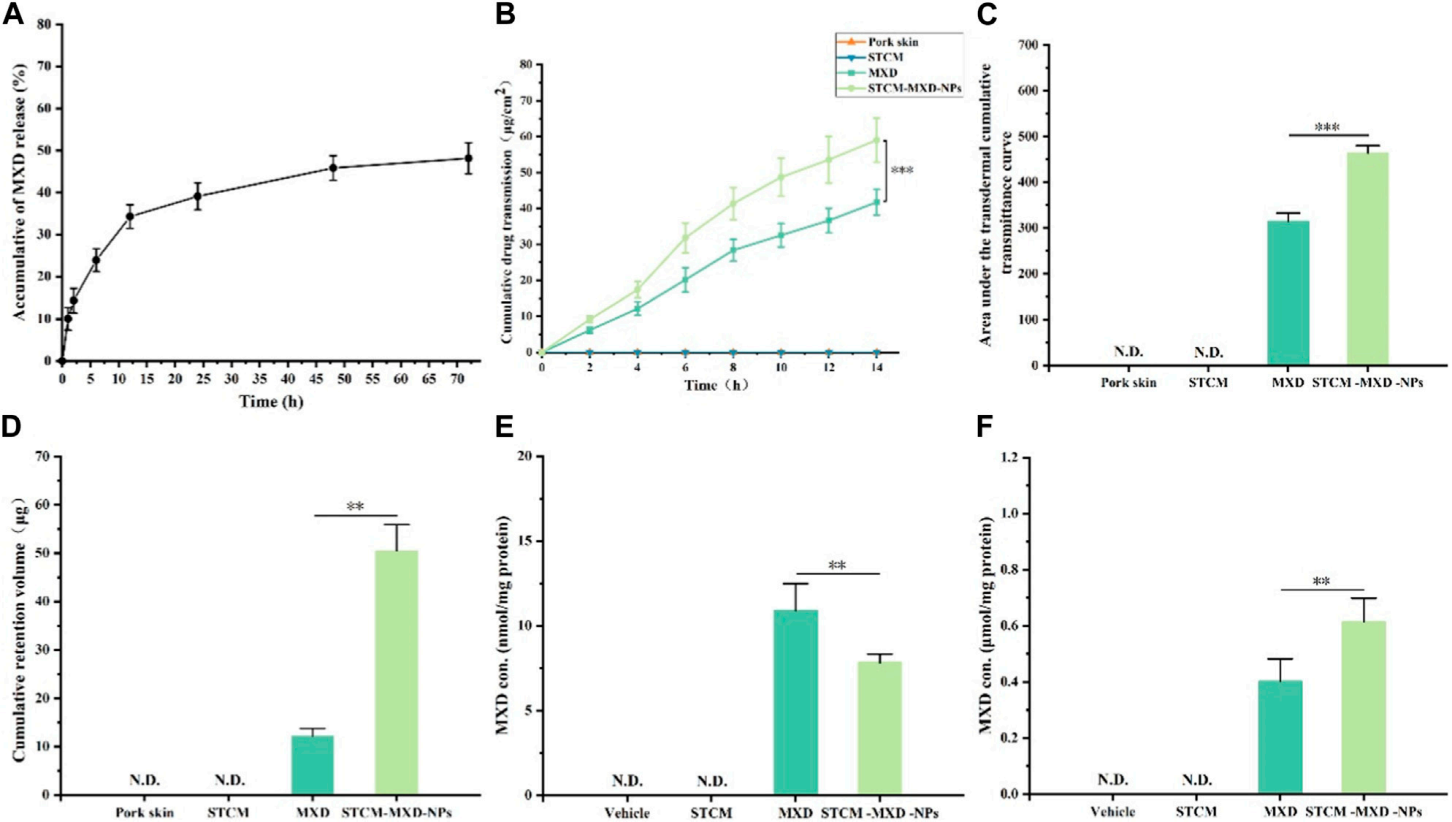
Drug-carrying properties of STCM-MXD-NPs. **(A)** Cumulative release profile of STCM-MXD-NPs. **(B)** Transdermal cumulative transmittance of MXD and STCM-MXD-NPs. **(C)** The area under the transdermal cumulative transmittance curves for MXD and STCM-MXD-NPs. **(D)** Retention of MXD and STCM-MXD-NPs. **(E)** MXD content in the skin tissue of mice. **(F)** MXD content in mouse hair bulb tissue. Key: *, *p* < 0.05; **, *p* < 0.01; ***, *p* < 0.001.

The *in vitro* release experiments of STCM-MXD-NPs are shown in Figure 3A. MXD was slowly released from the hMSC membranes, with a cumulative release percentage of about 48.15% within 72 h, and it continued to be slowly released after 72 h.

### STCM-MXD-NPs have enhanced transdermal capabilities

Figures 3B, C show that the 14 h cumulative amount of MXD transmitting through the skin was 41.73 ± 3.57 and 59.03 ± 6.14 μg/cm^2^ for MXD and STCM-MXD-NPs, respectively, showing sustained MXD transmission through the experimental pig skins. Therefore, the STCM-MXD-NPs have a more potent transdermal ability than MXD.

### STCM-MXD-NPs increase intradermal MXD retention

Figure 3D shows that after 14 h of transdermal absorption, MXD retention in the skin was 12.21 ± 1.56 μg with MXD and 50.46 ± 5.48 μg withSTCM-MXD-NPs. The intradermal MXD retention was higher in the STCM-MXD-NP group than in the MXD group at the same drug concentration.

### MXD in skin and hair bulb tissue

Addressing how to deliver more MXD to the hair bulb is vital in treating alopecia. In order to compare the utilization of MXD and STCM-MXD-NPs, we examined the amount of MXD in skin tissues and hair bulbs. The amount of MXD in skin tissues of the STCM-MXD-NP group was 71.72% that of the MXD group. However, the amount of MXD in hair bulbs was 1.53 times higher in the STCM-MXD-NP group than in the MXD group (Figures 3E, F).

### Effects of STCM-MXD-NPs on hair growth in mice

**FIGURE 4:**
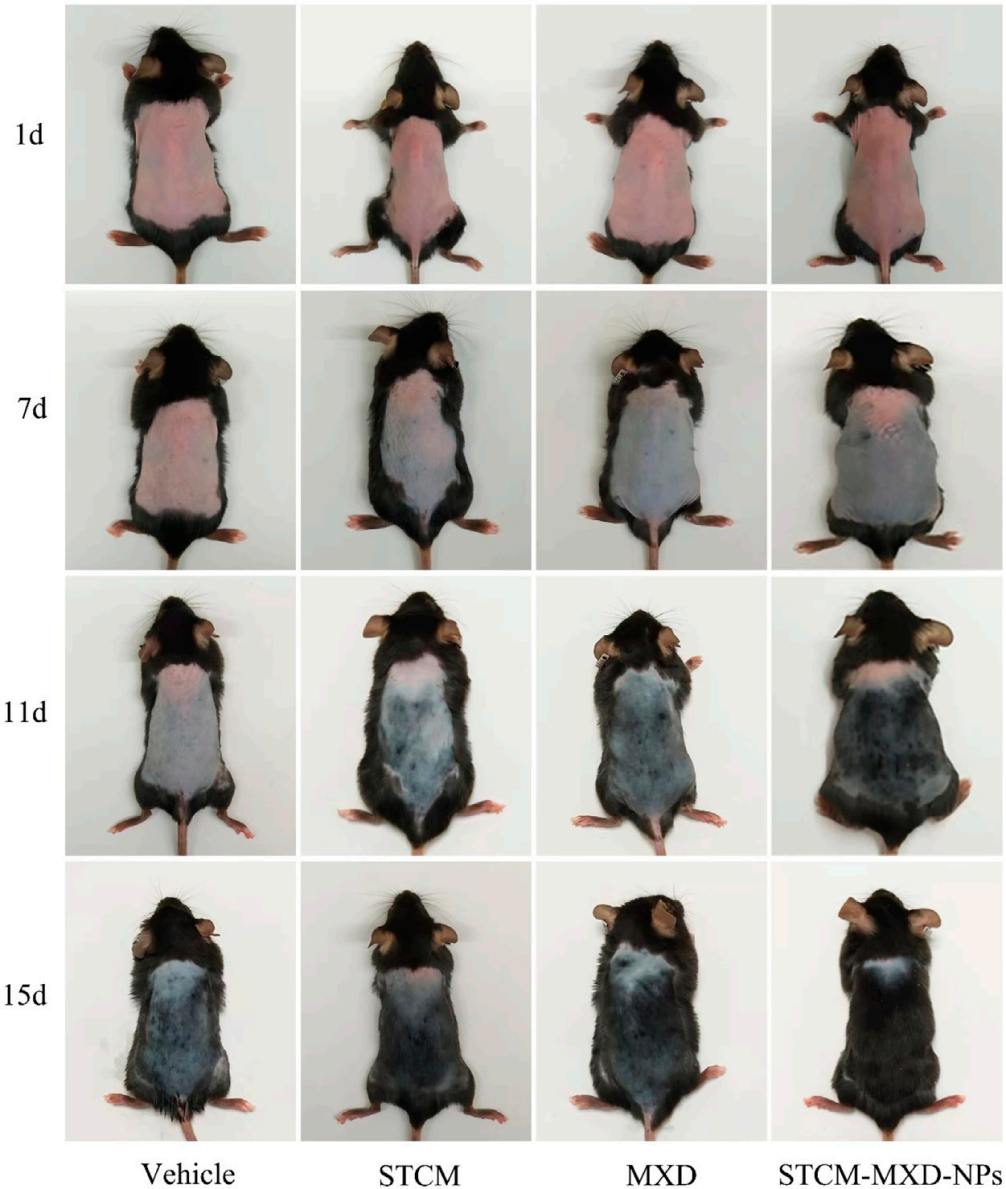
Comparison of hair growth in the different groups of C57BL/6J mice. The dorsal skin was imaged on days 1, 7, 11, and 15 of administering the treatment to the mice. Each 7-week-old male C57BL/6J mouse was shaved to remove dorsal skin hairs, and 200 μL of PBS, STCM, MXD, or STCM-MXD-NPs was topically applied to the depilated area on the back of the mice each day for 15 days.

In order to evaluate the effects of STCM-MXD-NPs on hair development and the number of hair follicles in mice, we subjected the back skin of mice to depilation treatment. Figure 4 shows that the skin on the back of mice in all groups was pink, with no redness or swelling and apparent no rupture. On days 1–7, the skin color of the back of the mice did not change significantly in the Vehicle group, was light grey in the STCM group, was grey in the MXD group, and mostly gradually changed from pink to dark grey in the STCM-MXD-NPs group.

On days 8–11, the skin on the back of mice only turned grey in the Vehicle group, with no hair outgrowth. In contrast, only a tiny amount of hair outgrowth was visible on the backs of mice in the STCM group, and a small amount of hair outgrowth was visible on the backs of mice in the MXD group. However, the backs of the mice in the STCM-MXD-NP group were darker in color, and most of the hairs were outgrown.

On day 15, only a small amount of hair growth was evident in the Vehicle group, while hair growth was seen in the STCM group. Much hair growth was evident in the MXD and STCM-MXD-NP groups. However, the hair was lighter in color in the MXD group than in the STCM-MXD-NP group, where the hair was mainly black and glossy. There were no symptoms of skin irritation, such as redness, swelling, desquamation, and ulceration, at the site of the back of the skin.

**FIGURE 5:**
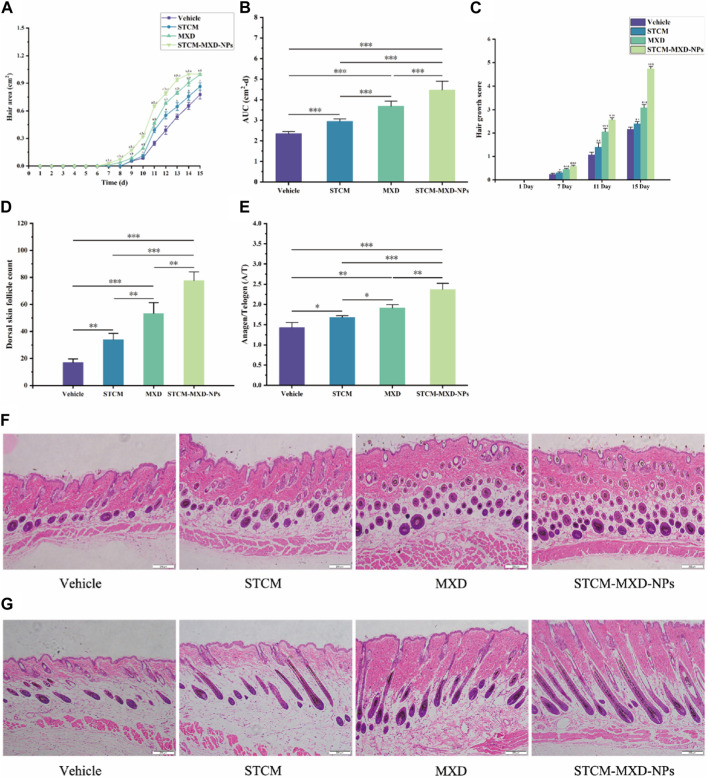
Comparison of hair growth in the different groups of C57BL/6J mice. **(A)** Comparison of dorsal skin growth areas. **(B)** Comparison of AUC of dorsal skin growth areas quantified using ImageJ software (*n* = 6). **(C)** Comparison of hair growth scores assessed using scoring metrics (0 = no growth, 1 = 0–20% growth, 2 = 20–40%, 3 = 40–60%, 4 = 60–80%, 5 = 80–100%). **(D)** Comparison of the number of hair follicles in the dorsal skin. **(E)** The A/T ratio of follicles in the dorsal skin. **(F,G)** HE staining of the dorsal skin (×100 magnification). Key: *, *p* < 0.05; **, *p* < 0.01; ***, *p* < 0.001.

In addition, we evaluated the hair growth rate of mice in each group (Figures 5A–C). The hair growth areas of mice in the STCM, MXD, and STCM-MXD-NP groups were 1.25, 1.57, and 1.90 times larger than that of the Vehicle group, respectively, with that of the STCM-MXD-NP group also being 1.21 times larger than that of the MXD group. In addition, the STCM-MXD-NP group showed a significant increase in hair growth scores after day 7 compared with the remaining groups. The STCM-MXD-NPs showed significantly better efficacy than MXD, significantly promoting hair growth.

Then, we assessed the number of hair follicles and the cycle of hair follicles in the dorsal skin of the mice. After 15 days of treatment, the number of hair follicles was significantly higher in the STCM-MXD-NP group than in the other groups. In addition, their hair length was longer, their hair follicles and the endogenous sheaths were well-developed and enriched with melanin particles, their hair follicles grew to the deeper layers of the subcutaneous tissues and sebaceous glands, and their hair follicles were in the anagen phase more often. The follicles of mice in the MXD group had fewer melanin granules than those in the STCM-MXD-NP group. Hair follicles and inner root sheaths were generally developed in the STCM group, and the bulb of hair follicles was more shallowly located. In addition, the number of hair follicles and the A/T hair follicle ratio were significantly higher in the STCM-MXD-NP group than in the MXD group (Figures 5D–G).

### STCM-MXD-NPs upregulate hair growth factor expression in C57BL/6J mice

**FIGURE 6:**
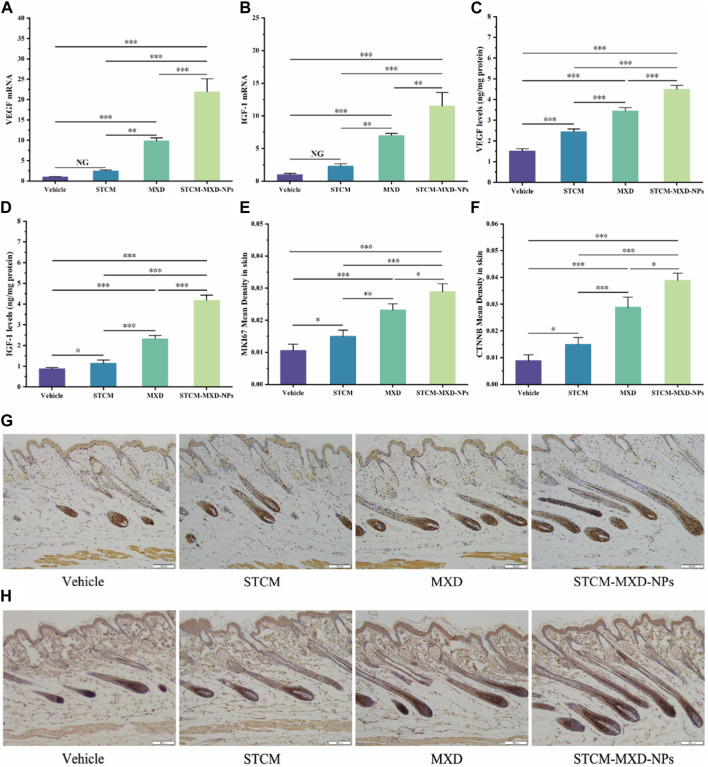
Comparison of hair growth factor expression in skin tissues of the different groups of C57BL/6J mice. **(A)** *Vegf* mRNA expression in hair bulb tissues. **(B)** *Igf-1* mRNA expression in hair bulb tissues. **(C)** VEGF protein expression in hair bulb tissues. **(D)** IGF-1 protein expression in hair bulb tissues. **(E)** Comparison of mean optical density values for MKI67 in the dorsal skin. **(F)** Comparison of mean optical density values for CTNNB in the dorsal skin. **(G)** MKI67 expression in the dorsal skin tissues. **(H)** CTNNB expression. Key: *, *p* < 0.05; **, *p* < 0.01; ***, *p* < 0.001.

We evaluated the effects of STCM-MXD-NPs on the mRNA and protein expression of VEGF and IGF-1 in the hairballs of mice (Figures 6A–D). Their mRNA and protein levels were significantly higher in the STCM-MXD-NP group than in the other groups. The *Vegf* and *Igf-1* mRNA levels were 2.22 and 1.64 times higher in the STCM-MXD-NP group than in the MXD group, and the VEGF and IGF-1 protein levels were 1.31 and 1.80 times higher in the STCM-MXD-NP group than in the MXD group.

The more vigorous the cell proliferation, the more pronounced the MKI67 expression and the deeper the hair follicle staining. After paraffin sections of mouse skin tissues from all groups were immunohistochemically stained for MKI67, mice in the STCM-MXD-NP group showed the deepest hair follicle staining and the highest MKI67 expression. The MKI67 expression in the STCM-MXD-NP group was 1.25-fold higher than in the MXD group, 1.93-fold higher than in the STCM group, and 2.74-fold higher than in the Vehicle group (Figures 6E, G).

CTNNB is important in promoting hair follicle cell proliferation and growth. It is strongly expressed in the anagen phase of hair follicles and weakly expressed in the catagen and telogen phases. Immunohistochemical staining for CTNNB in paraffin sections of mouse skin tissues from all groups showed that the hair follicles of mice in the STCM-MXD-NP group stained darker (i.e., had the highest CTNNB expression), and more hair follicles were in the anagen phase. The CTNNB expression in the STCM-MXD-NP group was 1.35-fold higher than in the MXD group, 2.61-fold higher than in the STCM group, and 4.42-fold higher than in the Vehicle group (Figures 6F, H).

## Discussion

Alopecia is a chronic progressive disease, and many treatments are available. MXD is used as a first-line treatment for alopecia. However, it works slowly during treatment and may cause irritation, such as itching, redness, and scalp flaking. Finasteride may cause sexual dysfunction, such as decreased libido, erectile dysfunction, and ejaculatory dysfunction (Friedman et al., 2002; Joshi et al., 2023; Shadi, 2023). Therefore, research into novel therapies for alopecia is crucial.

Today, nanoparticle drug-carrying is an emerging direction for drug delivery, such as cell membrane-encapsulated NPs. With continuous nanomedicine technology development, nanoparticle drug-carrying systems have been widely used in clinics (Tang et al., 2012). MSCs can self-renew and differentiate, showing low immunogenicity, promoting tissue repair, and having a robust immunomodulatory effect while being safe and non-toxic. Membrane-encapsulated NPs have excellent pharmacological activities, such as promoting pain relief and wound healing (Sotiropoulou et al., 2006; Naji et al., 2019; He et al., 2020; Zhou et al., 2020). MSC membrane-encapsulated NPs can improve drug delivery by effectively targeting specific regions and enhancing drug bioavailability. Therefore, stem cell membrane nano-loaded controlled-release systems have a potential role in delivering MXD to promote hair growth (Kalayeh et al., 2023).

This study synthesized STCM-MXD-NPs with a particle size of 181.4 ± 5.98 nm. We found that they not only had a better slow-release effect than conventionally delivered MXD, with MXD slowly released from the membrane of the encapsulated hMSCs to act continuously on the skin follicle tissues, but also had better transdermal properties and intradermal MXD retention. We have been studying how to deliver MXD more efficiently into the hair bulb tissue to better treat hair loss. Therefore, we investigated the delivery pathway of STCM-MXD-NPs and whether it is efficient to deliver MXD into the hair bulb through the hair follicle. We found that the MXD content in the hair bulb tissues was 1.53 times higher in the STCM-MXD-NP group than in the MXD group. However, the MXD content in the skin tissues in the STCM-MXD-NP group was only 71.72% that in the MXD group. These results suggest that the STCM-MXD-NPs more efficiently deliver MXD into the hair bulb, increasing the efficacy and having a better hair growth-promoting effect.

Next, we assessed the effects of STCM-MXD-NPs on hair growth. We used C57BL/6J mice as a model for hair follicle regeneration because melanin synthesis in hair follicle melanocytes coincides with the hair growth cycle, and the expression and activity of melanogenesis-associated proteins correlate with the hair cycle in mice (Slominski et al., 1994). The hair growth of C57BL/6J mice is cyclic. Hair follicles show the greatest proliferation in the anagen phase, synthesizing and secreting the most melanin. The skin is black, and the hair follicle grows actively. Their proliferation ability is weak in the catagen phase, synthesizing little melanin. The skin is grey, the hair follicle bulb begins to atrophy, and onion-like changes can appear. The follicle stops proliferating during the telogen phase, and melanin production stops. The skin is pink, the follicles are in a state of atrophy, and the hair easily falls off (Müller-Röver et al., 2001).

The mice in the STCM-MXD-NP group had the earliest darkening of the skin on their back, indicating that the hair in this group had entered the anagen phase earlier, and the AUC for hair growth area was 1.21 times higher in this group than in the MXD group. The number of hair follicles was significantly higher in the STCM-MXD-NP group than in the other groups. Not only were their hair follicles and endogenous sheaths well-developed, but they also showed the most abundant melanin granules. Their hair follicles also grew into the deeper layers of subcutaneous tissues and sebaceous glands, were more often in the anagen phase and least often in the telogen phase. The STCM-MXD-NPs effect in promoting hair growth may be mediated through the transition of hair follicles from the telogen phase to the anagen phase in C57BL/6J mice, stimulating their early embryonic development process and playing an important role in hair follicle development. The STCM-MXD-NPs even prolonged the anagen phase of hair follicles in mice, indicating they have a significantly higher potency than MXD.

Finally, we examined changes in the expression levels of proteins involved in hair growth. Kim et al. (2013) found that VEGF and IGF-1 expression levels were associated with hair growth and that IGF-I promotes hair growth by activating cells at the hair follicle, prolonging the anagen phase of the hair growth cycle (Park et al., 2007; Angunsri et al., 2011). VEGF regulates hair follicle stem cell development and differentiation. Its expression is upregulated during the anagen phase of the hair follicle (Li et al., 2012; Bak et al., 2018). VEGF is also an important factor in angiogenesis, which promotes hair growth by inducing tissue neovascularisation and generating a better microenvironment for hair regeneration by providing sufficient blood supply during development. However, VEGF and its receptor are only expressed in hair follicles during the anagen phase. VEGF expression is significantly reduced in the catagen and telogen phases. The vascularity of the perifollicular area is significantly increased when hair is in the anagen phase. Angiogenesis is decreased in the catagen and telogen phases. Therefore, perifollicular angiogenesis is closely related to VEGF expression (Leung et al., 1989; Yano et al., 2001; Detmar et al., 1994; Brown et al., 1995a; Brown et al., 1995b; Bi et al., 2019).

MKI67 is a characteristic protein expressed by proliferating cells. It marks the beginning of hair follicle stem cell division and the transition of hair follicles from the telogen phase to the anagen phase. It can be expressed incrementally during the anagen phase of the hair follicle, with a significant decrease in expression during the telogen phase (Liu et al., 2022).

CTNNB is also one of the most important proteins involved in hair growth. It is expressed periodically during the hair follicle growth process, with strong expression in the early hair follicle anagen phase and weak expression in the catagen and telogen phases. Therefore, it can regulate the hair’s growth cycle, inducing the anagen phase and promoting the transition of the hair into the anagen phase (Kikuchi, 2000; Xie et al., 2003; Grumolato et al., 2010; Chen et al., 2019). In addition, Wnt/CTNNB cell signaling also activates hair follicle stem cells and regulates hair follicle formation, which is essential for promoting hair follicle cell proliferation and growth. Moreover, CTNNB is closely related to melanoblast proliferation, migratory differentiation, and pigmentation during the follicular stage of hair follicle development (Goding, 2000; Aoki et al., 2003; Carreira et al., 2006; Karafiat et al., 2007; Bellei et al., 2011; Lim and Nusse, 2013).

In order to investigate the signaling mechanism of STCM-MXD-NPs in inducing the anagen phase, this study measured VEGF and IGF-1 expression levels by real-time qPCR and ELISA and examined their expression patterns by immunohistochemistry with MKI67- and CTNNB-specific antibodies. We found that STCM-MXD-NPs significantly upregulated the mRNA and protein levels of VEGF and IGF-1, suggesting that higher MXD content in hairballs caused higher VEGF and IGF-1 expression levels. In addition, the immunohistochemical results showed that the MKI67 and CTNNB expression levels were significantly higher in the STCM-MXD-NP group than in all other groups. These findings indicate that the growth-promoting effect of STCM-MXD-NPs on hair follicles could activate their development through the CTNNB pathway, stimulating them to enter the anagen phase or promoting the prolongation of the hair’s anagen phase so that most follicles were in the anagen phase.

In summary, the STCM-MXD-NPs promoted hair growth better than traditional MXD administration in C57BL/6J mice. The mechanism may be related to the fact that STCM-MXD-NPs have better transdermal properties and slower release, enabling them to continuously target the skin follicles to maintain their efficacy, and that MXD has a high storage capacity in the hair bulb tissue. In addition, STCM-MXD-NPs promoted VEGF, IGF-1, MKI67, and CTNNB expression, regulating the hair follicle growth cycle, inducing follicle transition into the anagen phase, and promoting neovascularisation, thereby promoting hair growth. This treatment aims to enable a potentially new and better way to treat alopecia and increase clinical research on alopecia.

## Conclusion

We designed MXD delivered via a stem cell membrane delivery controlled release system and showed that STCM-MXD-NPs could deliver MXD into the hair bulbs via the hair follicles and had a therapeutic efficiency on hair growth in C57BL/6J mice was higher than that of ordinary MXD. The skin toxicity of STCM-MXD-NPs prepared in this experiment may be very low because no organic solvents (propylene glycol and ethanol) were used in the whole preparation process, and no skin irritation symptoms, such as redness, swelling, desquamation, and breakage, were observed in the dorsal skin of mice in the STCM-MXD-NPs group in the experiment, which proves that the skin toxicity of STCM-MXD-NPs may be very low. These advantages of STCM-MXD-NPs provide important information and reference basis for exploring new therapeutic drugs for Alopecia patients.

## Data Availability

The raw data supporting the conclusion of this article will be made available by the authors, without undue reservation.
