# Supplementary material for: Minoxidil delivered via a stem cell membrane delivery controlled release system promotes hair growth in C57BL/6J mice
Source: Front Bioeng Biotechnol. 2024 Jan 8;11:1331754. doi: 10.3389/fbioe.2023.1331754 (PMC10800965; doi:10.3389/fbioe.2023.1331754)
Supplement: Supplementary file 1 [file DataSheet1.docx]

Supplemental data

Supplemental data 1

Table 1. Encapsulation rates of STCM-MXD-NPs.

| Serial number | MXD concentration (mg/mL) | Encapsulation method | Encapsulation efficiency (%) |
| --- | --- | --- | --- |
| 1 | 1 | Ultrasonic | 15.56% |
| 2 | 2 | Ultrasonic | 27.52% |
| 3 | 3 | Ultrasonic | 35.75% |
| 4 | 4 | Ultrasonic | 42.58% |
| 5 | 5 | Ultrasonic | 54.81% |
| 6 | 6 | Ultrasonic | 62.56% |
| 7 | 7 | Ultrasonic | 57.57% |
| 8 | 8 | Ultrasonic | 52.25% |
| 9 | 9 | Ultrasonic | 48.72% |

Supplemental data 2


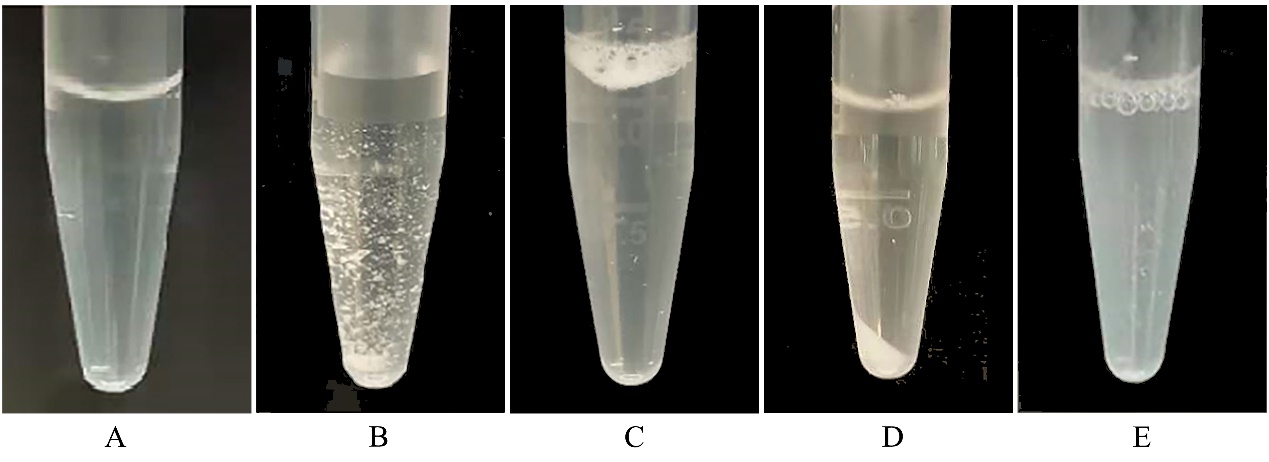


**Figure 1.** Solubility of MXD and hMSC membrane-loaded MXD NPs.

(A) MXD in ethanol solution. (B) MXD in PBS solution. (C) hMSC membranes in PBS solution. (D) hMSC membranes loaded with finished MXD NPs. (E) hMSC membranes loaded with MXD NPs in PBS solution.

Supplemental data 3


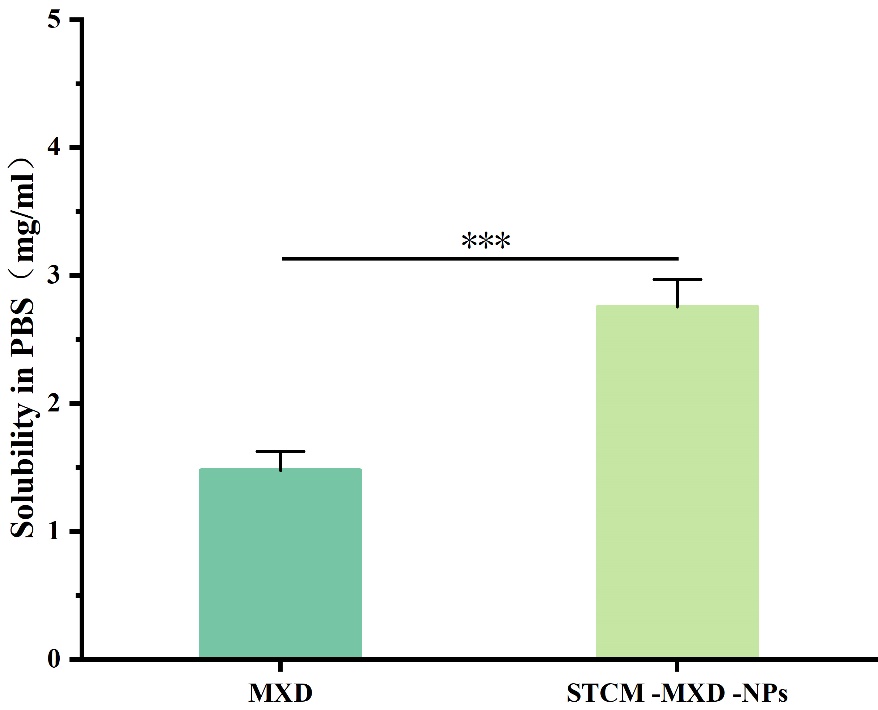


**Figure 2.** Solubility of MXD and STCM-MXD-NPs in PBS.

Key: *, p < 0.05; **, p < 0.01; ***, p < 0.001.
